# An implantable wireless tactile sensing system

**DOI:** 10.21203/rs.3.rs-2515082/v1

**Published:** 2023-02-03

**Authors:** Lin Du, Han Hao, Yixiao Ding, Andrew Gabros, Thomas C. E. Mier, Jan Van der Spiegel, Timothy H. Lucas, Firooz Aflatouni, Andrew G. Richardson, Mark G. Allen

**Affiliations:** 1 Department of Electrical and Systems Engineering, School of Engineering and Applied Science, University of Pennsylvania, Philadelphia, PA 19104, USA.; 2 Department of Neurosurgery, Perelman School of Medicine, University of Pennsylvania, Philadelphia, PA 19104, USA.; 3 Departments of Neurosurgery and Biomedical Engineering, The Ohio State University, Columbus, OH 43210, USA.

## Abstract

The sense of touch is critical to dexterous use of the hands and thus an essential component to efforts to restore hand function after amputation or paralysis. Prosthetic systems have focused on wearable tactile sensors. But wearable sensors are suboptimal for neuroprosthetic systems designed to reanimate a patient’s own paralyzed hand. Here, we developed an implantable tactile sensing system intended for subdermal placement. The system is composed of a microfabricated capacitive force sensor, a custom integrated circuit supporting wireless powering and data transmission, and a laser-fused hermetic silica package. The miniature device was validated through simulations, benchtop testing, and ex vivo testing in a primate hand. The sensor implanted in the fingertip accurately measured skin forces with a resolution of 4.3 mN. The output from this novel sensor could be encoded in the brain with microstimulation to provide tactile feedback. More broadly, the materials, system design, and fabrication approach establish new foundational capabilities for various applications of implantable sensing systems.

## Introduction

Skin is endowed with a variety of tactile mechanoreceptors that play an invaluable role in sensing our environment and guiding movement. A lack of tactile feedback severely limits dexterity of both biological (1) and biomimetic robotic systems (2). Thus, multiple approaches have been used to realize artificial tactile sensors for wearable electronics, prosthetics, and robotics (3–5). In general, these tactile sensors can be classified according to the sensing principles employed, such as capacitive (6–9), piezoresistive (10–17), and magnetic field-based sensing technologies (18,19). These sensors have been embedded in flexible and stretchable substrates for applications such as healthcare monitors (20), prosthetic skins (21), patient rehabilitation (22), electronic skins (6), and robotic skins (23). Wearable designs such as sensorized gloves or skin-attached electronics are commonly adopted for these sensor systems.

However, wearable sensors are suboptimal for applications in which the goal is to restore a sense of touch to biological skin, as in the case of paralysis. Paralysis disrupts both motor and somatosensory signals between the brain and body. Brain-machine interface (BMI) technology has been used to implement brain-controlled muscle stimulation to reanimate a paralyzed limb (24, 25). This strategy can restore volitional hand movement in humans with tetraplegia (26, 27). But tactile sensing in the palm and fingertips that is critical for dexterous manipulation is still lacking. One could consider augmenting such systems with tactile sensors coupled with appropriate neural stimulation to artificially encode the sense of touch (28). Although wearable sensors are a potential candidate for supplying this tactile functionality noninvasively, they have several disadvantages. Skin-attached devices have limited longevity due to epidermal turnover and environmental interference. Wearables place often ill-fitting material between the skin and grasped object, thus altering the natural interface. Finally, bulky sensorized gloves could place undue burden on activated muscles already prone to fatigue due to unnatural recruitment (29).

An alternative approach to resolve these issues is an implantable tactile sensor system. Implantable microelectromechanical systems (MEMS) sensors and actuators have been adopted to monitor human health conditions, improve quality of life, and save lives (30–32). However, long-term implantable technology is extremely challenging due to the requirements on hermeticity, biocompatibility, as well as a proper form factor (33). The system should attain long-term hermeticity to provide any enclosed electronic circuitry with protection from the harsh environment of the human body. Simultaneously, the packaging material should be biocompatible and small enough to fit within the target location; and ideally should also support communication means so data (and optionally power) can be transferred to and from the sensor.

Over the past several decades, many implantable biomedical systems have been approved by the Food and Drug Administration (FDA) and achieved real-world healthcare applications. Examples include cardiac pacemakers for stimulating cardiac contraction; heart failure monitoring systems (e.g. CardioMEMS) for measuring arterial pressure; glucose monitoring systems for measuring glucose in the interstitial fluid; cochlear implants for restoring hearing; spinal cord stimulators for alleviating chronic pain; and deep brain stimulators for treating movement disorders such as essential tremor and Parkinson’s disease (33,34). Typically, these systems are composed of an implantable sensor or actuator, an energy source for powering the electronics (either a battery or a wireless power transmission system), as well as signal communication electronics. If wireless transmission of power or data is involved, an external device is needed for communication with the implanted electronics for signal processing and display. To satisfy the long-term hermicity and biocompatibility requirements, materials such as titanium (Ti), alumina (Al2O3), and fused silica (SiO2) are the most popular choices. For example, pacemakers employ Ti cans (encapsulating battery and circuitry) with glass-sealed feedthroughs for connection of signal transfer (35). However, metal is not transparent at radio frequencies (RF). Therefore, additional means, such as providing an RF-transparent window, are typically required to communicate with such devices. Fused silica, which is transparent to optical and radio frequency signals, largely chemically inert, and biocompatible, is an attractive choice and is currently used in the CardioMEMS system (36). Further, the availability of this material in wafer form facilitates its use in standard micromachining processes such as lithography, wafer bonding, and laser machining.

Here, we utilized silica-based MEMS technology to develop the first implantable tactile sensor that is fully wireless, obviating the need for subdermal tunneling of electrical leads across joints (37). This sensor system is intended to be combined with sensor-controlled brain stimulation (38) and brain-controlled muscle stimulation (26, 27) to provide closed-loop hand reanimation in paralyzed subjects. The addition of tactile feedback to reanimation strategies would be a substantial step towards a clinical BMI, allowing the thousands of newly paralyzed individuals each year to regain functional independence.

## Results

### System design

**Fig. 1.**
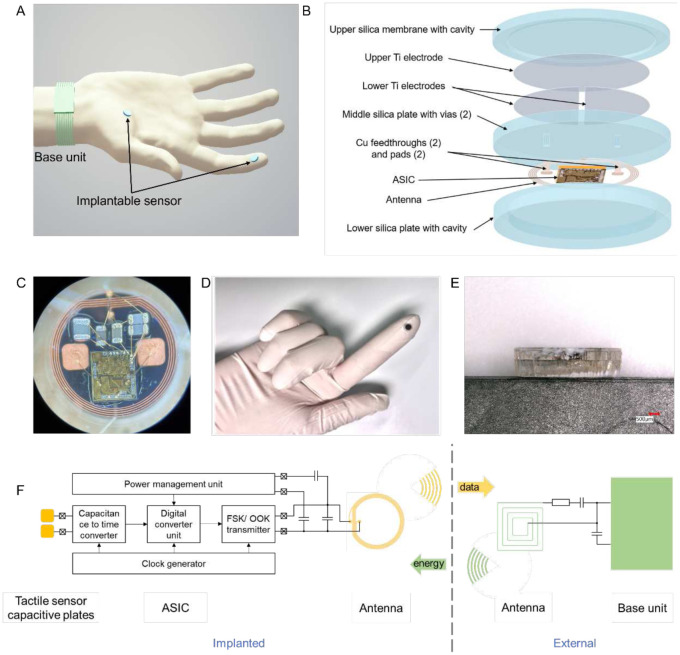
Implantable wireless tactile sensing system. (**A**) Illustration of the tactile sensing system wirelessly monitoring forces acting on the fingertip and palm. The implantable sensing system integrates capacitive force detection, signal processing, and customized wireless data and power transceiver interfaced to a battery-powered wearable base unit. (**B**) Exploded view of the implantable sensor, which contains five layers: an upper silica membrane, capacitive double plates, a middle silica plate (with vias, feedthroughs, and pads), ASIC with antenna and electronic components, and a lower silica plate. (**C,D**) Images of the front and back of the sensor. (**E**) Side view of the hermetic microsystem with fused silica plates. (**F**) System-level block diagram.

The envisioned implantable, wireless tactile sensing system combines miniaturized, biocompatible force sensors that could be implanted under the skin at any desired location, such as the palm or fingertips, with wireless power and data links to a battery-powered base unit worn on the wrist, dorsum of hand, or fingernail (Fig. 1A). To realize this system, we developed an integrated implantable platform, fully encapsulated with biocompatible and hermetic silica layers and consisting of three main components (Fig. 1B). First, there is a parallel plate capacitive force sensor containing an upper silica plate and a middle silica plate with a cavity in between. Underneath the upper pate, there is a circular upper electrode. On top of the middle plate, there are two semi-circular lower electrodes electrically connected to pads on the underside of the middle silica plate by means of feedthroughs. Second, there is a miniaturized, wireless transfer module including an application-specific integrated circuit (ASIC), capacitors, an antenna coil, and bond wires. The module can communicate power and data between the implantable sensor and wearable base unit (Fig. 1C). Third, there is a three-layer package fully encapsulated with biocompatible and hermetic silica material based on a laser-assisted fusion bonding technology (39, 40). The upper layer provides a flexible pressure sensing membrane. The middle layer, shared by the sensor and the electronics module, supports electronic connections with pads and vias. The lower layer with the cavity provides space and protection for the electronics. Fabrication details are provided in the Materials and Methods and Figure S1. Figure 1D shows the frontside view of the implantable system, with the black circular upper electrode seen through the transparent upper silica plate. The thickness of the three fused silica layers were 200 μm for the upper plate, 500 μm for the middle plate, and 1 mm for the lower plate (Fig. 1E).

The wireless transfer module, operating at 35 MHz, simultaneously achieved energy transfer and data communication based on magnetic human body communication (mHBC) (41). Figure 1F shows the block diagram of the implemented communication interface between the implantable sensing system and the wearable base unit. The implanted ASIC consists of a power management unit (PMU), a capacitance to time converter (CTC), a clock generator, a digital control unit, and the data transmitter. The PMU rectifies the received energy signal from the base unit and provides regulated voltage supplies for other blocks. It also monitors the sensor input power level, which is then used in the wireless power measurement feedback system to compensate for gesture changes and process-voltage-temperature variations. As we reported previously, the ASIC achieves a resolution of 3.4 fF for a 18 pF input capacitance, consuming 110.3 μW (42, 43). Specifications for the system are summarized in Table 1.

### Sensor design and simulation

**Fig. 2.**
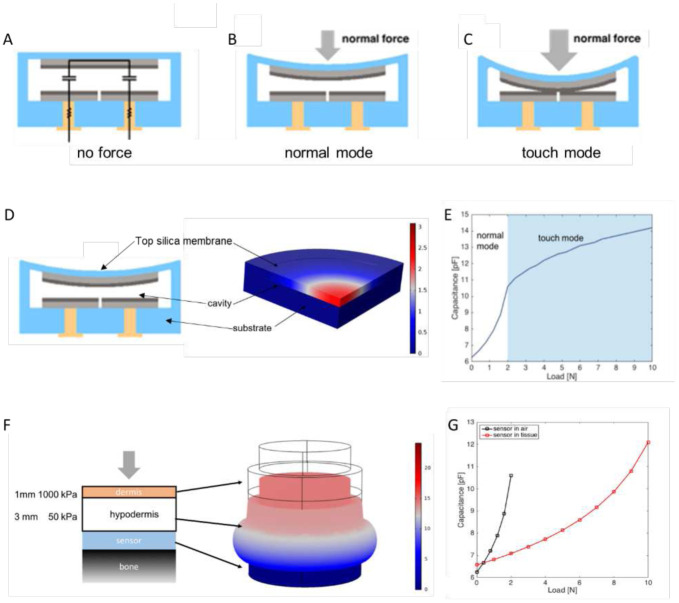
Sensor operation principle and finite element simulation results. **(A)** Sensor equivalent circuit diagram: two semi-circular capacitors connected in series by the upper electrode with a floating potential. **(B)** Sensor in normal mode operation. **(C)** Sensor in touch mode operation. **(D)** Simulation model of static forces applied directly to the sensing membrane. **(E)** Direct force model results: sensor capacitance vs. applied load. **(F)** Simulation model of static forces applied to the surface of skin overlying the implanted sensor. Skin modeled as two elastic layers (dermis, hypodermis). **(G)** Simulated sensor response to tactile forces when implanted below skin (red) compared to the response to direct forces on the sensor membrane (black).

The operation principle of the capacitive sensor is illustrated in Figure 2. When there is no force applied to the sensor, the upper silica plate, with a floating potential upper electrode, is parallel with the middle silica plate, containing two lower electrodes (Fig. 2A). In the presence of normal force acting on the sensor membrane, the upper plate deflects toward the middle plate, decreasing the interelectrode distance and increasing the capacitance between the two lower electrodes (Fig. 2B). As the upper plate deflects further with increasing force, the upper electrode may touch the lower electrodes, resulting in touch-mode operation (Fig. 2C). To prevent electrical shorting during touch mode operation, the electrodes can be coated with a thin insulating dielectric such as an oxide layer. Note that even in touch mode, the interelectrode capacitance will continue to increase with increasing external load, as the upper sensor plate will continue to deform and conform to the lower plate electrodes.

To quantitatively evaluate the sensor operation principle, a COMSOL Multiphysics simulation using the solid mechanics module and the AC/DC electrostatics module was performed (Fig. 2D). The loading procedure was simulated in a two-step fashion (Fig. S2). In the first step, the solid mechanics module was applied to study the deflection of the upper plate. In the second step, the solid mechanics module and electrostatics module were used to analyze the capacitance of the sensor under specific upper plate deflections. The capacitance of the sensor under each deflection profile could then be obtained. The simulation found that from 0 to 2 N, the sensor operates in normal mode (Fig. 2E). At 2 N, contact between the two plates occurs and touch mode begins. Above 2 N, the sensor continues to operate in touch mode and the capacitance increases more slowly.

For the intended tactile sensing application, the sensor microsystem would be implanted under the skin. Forces acting at the skin surface would be conveyed to the sensor membrane indirectly through the intervening tissue. To simulate these conditions, homogeneous elastic dermal and hypodermal layers were added to the simulation (Fig. S3). Due to the low Young’s modulus of the hypodermal layer, the deflection of the hypodermis was more significant than the deflection of the dermis in response to a normal force at the skin surface (Fig. 2F). Without lateral tissue constraints, the simulation found that there would be a 23% attenuation of the applied force due to tissue deformation (bulging), indicating that a 10 N pressure applied to the skin surface results in 2.3 N force applied directly on the sensor upper plate, with the corresponding attenuation of the sensor capacitance (Fig. 2G).

### Benchtop performance

**Fig. 3.**
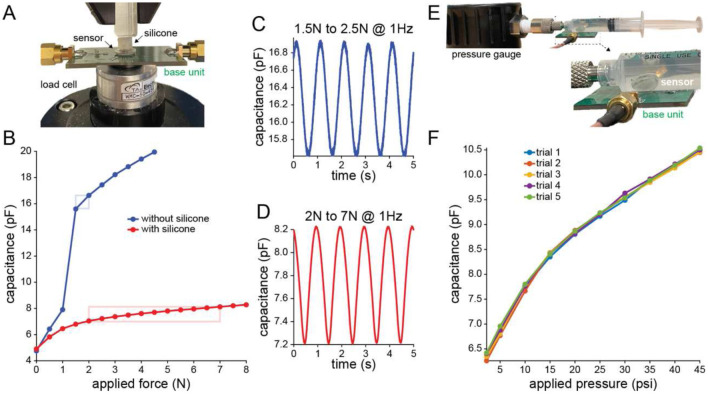
Benchtop sensor performance. **(A)** Direct compression testing setup, shown here with silicone layer mimicking skin overlying the sensor. **(B)** Sensor response to static forces applied directly to sensor membrane (blue) or through silicone layer (red). Boxes indicate the force range used for subsequent dynamic loading. **(C,D)** Sensor response to sinusoidal dynamic loading at 1Hz on the sensor membrane (blue) or through the silicone layer (red). **(E)** Hydrostatic testing setup, with the sensor placed in water within a syringe. **(F)** Sensor response to hydrostatic pressures

The force-capacitance relationship of the microfabricated sensor was initially assessed by applying static forces directly to the sensor membrane using a force-controlled test instrument. In accordance with our simulation, the wirelessly monitored sensor capacitance increased nonlinearly with normally-directed force. The capacitance of the sensor increased from 4.8 pF to 15.6 pF under loading from 0 N to a touch point at 1.5 N (Fig. 3B, blue). Once touch mode was achieved, the capacitance increased with reduced slope with further increases in applied force.

Next, as in the simulation, we assessed sensor performance under mechanical conditions mimicking implantation under the skin. A flat surface loading component was used to apply normal forces through a layer of prosthetic silicone to the sensor below (Fig. 3A). Overall, the sensor capacitance exhibited the same pseudo-parallel-plate behavior seen in direct force application. However, for a given change in sensor capacitance, a larger force was required when the silicone was present compared to not present, as predicted by our simulation. The capacitance of the sensor increased from 4.9 pF to 8.3 pF under loading from 0 to 8 N through the silicone (Fig. 3B, red).

Given that the sense of touch typically involves time-varying, rather than only static, forces acting on the skin, the dynamic performance of the sensor prototype was also assessed. To achieve a stable dynamic measurement, a minimum force of at least 1 N was required by the test instrument, putting the sensor near the transition from normal to touch mode in direct force application. Applying a 1 Hz sinusoidal direct force ranging from 1.5 to 2 N (touch mode) resulted in a corresponding sinusoidal change in capacitance in the range of 15.6 pF to 16.9 pF (Fig. 3C). While the static testing result indicated that the sensor capacitance was 15.6 pF at 1.5 N and 16.6 pF at 2 N, which is 0.3 pF higher, this was mainly due to the difficulty of applying a stable dynamic loading condition with this minimal loading. A similar dynamic test was performed in the case of applying forces through a layer of prosthetic silicone to the sensor. A 1 Hz sinusoidal force ranging from 2 to 7 N resulted in sinusoidal change in capacitance in the range of 7.2 pF to 8.2 pF (Fig. 3D). These values agreed well with the static force output capacitance of 7.0 pF at 2 N and 8.1 pF at 7 N.

Another important mode of operation for in vivo use is to sense hydrostatic pressure rather than direct compressive forces on the sensing membrane. To understand this mode of operation, the sensor was placed in a water-filled syringe connected to a pressure gauge (Fig. 3E). The capacitance of the sensor inside the syringe increased from 6.4 pF to 10.5 pF when pressure increases from 2.3 psi to 45 psi, which corresponds to 0.2 N to 3.9 N considering the dimension of the sensing membrane (Fig. 3F). In addition to demonstrating that the sensor can provide a reliable wireless measurement of hydrostatic pressure, this test showcased the watertight, hermetic silica package that we have previously shown to be capable of long-term use in vivo (44).

### Ex vivo performance

**Fig. 4.**
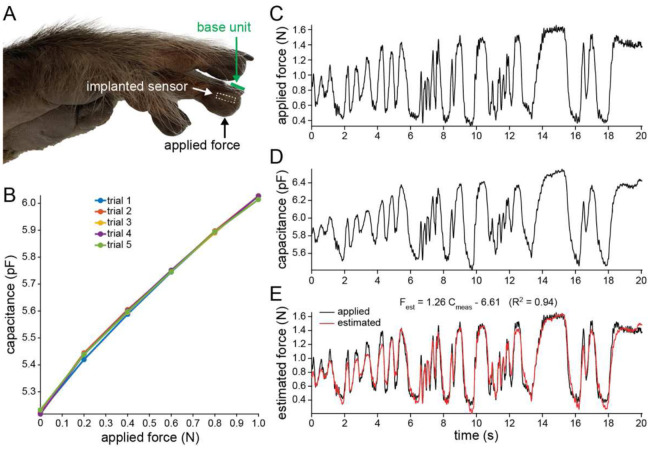
Ex vivo sensor performance. **(A)** Image of cadaver monkey hand after the wireless sensor was surgically placed in the indicated fingertip. The base unit was placed on the fingernail and static and dynamic forces were applied to the skin overlying the implanted sensor. **(B)** The sensor response to static, light tactile forces, showing sensitivity and repeatability. **(C)** Dynamic forces applied to the fingertip over 20 s. **(D)** The sensor response to dynamic forces. **(E)** Estimated force based on the linear transformation of sensor output compared to the applied force.

We performed a series of experiments to assess the device performance in conditions closely mirroring the intended tactile sensing application. The sensor was implanted into the fingertip of a fresh (not formalin-fixed) nonhuman primate hand obtained from an animal that had recently been euthanized for clinical reasons unrelated to this project. Through a small lateral skin incision, the back of the device was placed against the distal phalanx underneath the fingertip pulp and the skin was sutured closed. A custom-built, force-controlled motorized stage was used to apply small forces to the fingertip, in the range of physiological light touch (< 1N), while wirelessly recording the sensor response from above the fingernail (Fig. 4A). The sensor response to static forces was repeatable and nearly linear, with a sensitivity of 0.8 pF/N (Fig. 4B).

Time-varying forces were subsequently applied through manual indentation of the fingertip with a load cell recording applied forces (Fig. 4C). The sensor capacitance changes measured wirelessly by an external base unit closely followed the dynamic forces (Fig. 4D). A simple linear transformation of the sensor output could reliably estimate (R^2^ = 0.94) the applied dynamic forces (Fig. 4E). Together, the results show that the capacitive sensor system has a form factor suitable for subdermal implantation in the fingertip and can be used to faithfully estimate tactile forces for use in sensory restoration systems.

## Discussion

We developed a novel wireless capacitive force sensor intended for long-term operation within the body. Force transduction occurred through mechanical deformation of a hermetic fused silica package housing parallel plate electrodes and an ASIC that wirelessly communicated capacitance values to a wearable base unit. The device was validated through simulation, benchtop testing, and ex vivo testing in a primate hand.

The sensor microsystem was designed to function as an artificial mechanoreceptor for neuroprosthetic applications, sensing forces acting on the skin from an implant location under the skin. Given that the implanted sensor had a measured sensitivity of 0.8 pF/N and that the ASIC provided a capacitance resolution of 3.4 fF (43), we estimate that the force resolution of the device was 4.3 mN. For reference, low-threshold mechanoreceptors (LTMRs) in the skin and somatosensory cortical neurons three synapses downstream have an absolute response threshold of about 1 and 5 mN, respectively (45). The absolute threshold for perceptual awareness is similarly in this range (46). The perceptual just noticeable difference (JND, i.e., difference threshold) for the sense of touch asymptotes at about 10% for reference force levels above 2 N (e.g., minimum changes of 200 mN and 300 mN are perceived at a reference of 2 N and 3 N, respectively) (47). The JND increases as the reference force approaches absolute threshold (e.g., 50% JND, or 50 mN, at a reference of 100 mN) (48). Thus, the force resolution of our implantable sensor is approximately equal to the absolute threshold of natural touch and well below the JND at any reference force level. This ensures that for the intended application of artificial touch feedback, the performance will be limited by the encoding of sensor output into the brain with microstimulation (49) rather than by the sensor itself.

In contrast to force resolution, the spatial resolution of the sensing system (6 mm) is lower than that provided by natural skin mechanoreceptors. Slowly adapting and rapidly adapting LTMR subtypes have receptive fields as small as 2 mm and 0.5 mm diameter, respectively (50). However, for the application, it is unlikely that full biomimicry would be feasible, again due to limits in artificial encoding, nor needed functionally. A few well-placed sensors can dramatically improve grasping behaviors (38). That said, the current prototype was not optimized for size. The high temperature laser fusion bonding process for the hermetic silica package requires a minimum diameter of about 1.2 mm to thermally insulate an infinitely small ASIC placed at the center (30); even this minimum size could be reduced through optimization of laser parameters. The current ASIC size (1.37 mm × 1.18 mm) could be decreased and the passive elements surrounding the chip could be integrated to minimize the size above the laser fusion floor. Decreasing the diameter would decrease the sensitivity of the capacitive sensor, but this could be partially compensated by shrinking the gap between the capacitive plates in the microfabrication process, as well as reducing the thickness of the deflecting plate. Finally, decreasing the coil size would likely negatively impact wireless transmission range; further investigation would be required to understand this quantitatively.

Indeed, wireless transmission range was also not optimized in this work. The current device was demonstrated to function when implanted in the fingertip pulp with primary coil of the wearable base unit on the fingernail a few mm away. Although not yet capable of supporting an entirely wrist-worn base unit, even the current implementation would be beneficial as it would provide tactile sensing without the haptic interference inherent in wearable force sensors placed between the skin and grasped object (51). Future iterations of the device could increase the wireless range through increasing base unit power output, lowering ASIC power consumption, and taking better advantage of the mHBC channel (41, 43).

In practical use, the implanted sensor would be susceptible to atmospheric pressure changes that could be offset by barometric sensing in the base unit. In addition, periodic calibration could be obtained through grasping known weights and triggering a calibration routine in the base unit microcontroller. Regarding longevity in the body, our prior work encapsulating a humidity sensor in the same fused silica package found that the water vapor leakage rate was less than 4.6 × 10^−14^ atm•cm^3^/s (30). Based on a threshold water vapor concentration within the package of 5000 ppm and an air volume of 0.0012 cm^3^, the predicted viability of the encapsulated ASIC is 70.7 years. Future work will be needed to characterize the mechanical lifetime of the deformable package under repeated loading, as well as the effects of biofouling from the foreign body response to chronic implantation on sensor performance.

The materials, device designs, and fabrication approaches introduced here serve as foundations for implantable wireless sensing systems that can monitor forces and pressures inside the body for various other applications, such as intracranial pressure and bladder pressure. The minimized and smooth form factor of fused silica package promise its biocompatibility, transparency at radio frequency for wireless transmission, as well as transparency at optical frequency for other micro-opto-electro-mechanical systems (MOEMS).

## Materials and methods

### Fabrication of the upper plate

Schematic illustrations of the fabrication steps are shown in Figure S1. A first fused silica wafer, 20 mm × 20 mm square and 200 μm thick, is employed as the starting material for the upper plate. Positive photoresist SPR220–7.0 is spin-coated on the wafer with a speed of 500 rpm for 10 seconds and 3000 rpm for 50 seconds. Subsequently, the wafer is placed on a hot plate at 110 °C for 3 minutes for a soft bake. A lithography process is performed to expose a circular region of 4 mm in diameter with a mask pattern. The wafer with photoresist on top is then developed in MF-26A to define the pattern for a subsequent etching process. Afterward, reactive ion etching (RIE) is implemented to achieve a cylindrical cavity of 3.6 μm depth on the substrate wafer. Subsequently, the photoresist is stripped with acetone. To fabricate the upper electrode inside the cavity, photoresist S1805 is spray-coated and soft-baked at 115 °C for 90 seconds. Since the spray coater requires a 4-inch wafer for processing, the square shape wafer is attached to a 4-inch silicon wafer using crystal bond adhesive and detached during the soft bake. Afterward, lithography patterning for the circular shape upper electrode is performed. The wafer with photoresist on top is then developed in MF-319 to define the pattern for a subsequent deposition process. A 200 nm Ti layer and 30 nm SiO2 layer are evaporated and patterned using lift-off by acetone to complete the fabrication of the upper plate.

### Fabrication of the middle plate

A 4-inch fused silica wafer 500 μm in thickness is employed as the starting material for the middle plate (Fig. S1). To fabricate the connection pads and coil on the substrate, a seed layer comprising 30 nm Ti and 200 nm Cu is evaporated on the surface of the substrate wafer. Negative photoresist KMPR 1050 is spin-coated on the wafer with a speed of 500 rpm for 10 seconds and 4000 rpm for 30 seconds. Subsequently, the wafer is placed on a hot plate at 100 °C for 14.5 minutes for a soft bake. A lithography process is performed to expose the coil and pad regions. Then, the wafer is placed on a hot plate again at 100 °C for 3.5 minutes for post-exposure bake. The wafer with photoresist on top is then developed in SU-8 developer for 6 minutes to define the pattern for a subsequent etching process. Afterward, Cu pads and coils of 40 μm thickness are electrodeposited through the KMPR1050 photoresist mold at a current density of 10 mA/cm^2^ using a homemade Cu electroplating solution. The photoresist is then stripped with Remover PG. Seed layers are then sequentially removed with Cu etchant and Ti etchant to facilitate optical alignment in subsequent steps.

After pads and coil formation, fabrication proceeds for the feedthrough structure. Excimer laser rastering in high fluence mode is used to form feedthrough vias of size 250 μm × 250 μm until the vias reach the Cu pads on the bottom surface. The laser patterning induces a bump at the edge due to the redeposition of material during the ablation. To reduce the redeposition effect, a thin film of ProtectoLED (IPG Photonics) film is applied on the top of the substrate prior to the laser ablation and removed after the laser ablation. To fabricate the feedthrough structure, 30 nm thick Ti and 200 nm thick Cu seed layers are redeposited on the external surface of the wafer and passivated with nail polish which could achieve a thickness of tens of microns covering the pads and seed layers completely. During the laser ablation process above, Cu pads underneath the vias could be easily oxidized due to the heat. Therefore, the wafer is immersed in citric acid to remove Cu oxidation before the feedthrough electroplating process. Afterward, Cu feedthroughs approximately 350–450 μm in thickness are electrodeposited through the vias using a current density of 20 mA/cm^2^ using a commercial Cu electroplating solution. The passivation layer is then stripped with acetone. And seed layers are then sequentially removed with Cu etchant and Ti etchant. To deposit the electrodes and connect the electrodes to the feedthroughs, photoresist S1805 is spray-coated and soft-baked at 115 °C for 90 seconds. Since the spray coater requires a flat surface for vacuum, the middle plate with electroplating structures is attached to a 4-inch silicon wafer by crystal bond and detached during the soft bake. Afterward, lithography patterning for the two lower semi-circular shape electrodes is performed. The wafer with photoresist on top is then developed in MF-319 to define the pattern for a subsequent deposition process. A 400 nm thick Ti layer is sputtered at a chamber pressure of 7 mTorr on the substrate layer, forming both the electrodes on the lower surface and electrical connections to the feedthrough vias. The Ti layer is patterned using lift-off to complete the substrate fabrication.

### Fabrication of the lower plate

To provide a hermetic encapsulation of the electronics, a bottom plate with deep cavity of 600 μm is required. A two-step CO_2_ laser fabrication is adopted for the process (Fig. S1). To fabricate the cavity in the bottom plate, a 4-inch fused silica wafer 1 mm in thickness is employed and diced to 15 mm × 15 mm square shape by dicing saw. In the first step, the cavity fabrication process starts with a 5 mm in diameter circular shape CO_2_ laser rastering with a recipe of 100% power and 60% speed to fabricate a cavity of 8 μm deep. In the second step, a 4.6 mm in diameter circular shape rastering with a recipe of 100% power and 10% speed of 5 times is implemented to fabricate a cavity of 600 μm deep with a 5 μm lip which is concentric with the circular cavity in the first step. The lip introduced in the second step is lower than the cavity in the first step. Therefore, a cavity of 600 μm deep is accomplished to finish the fabrication for the bottom plate.

### Assembly of electronics and device

Assembly of the device started with a temporary bonding of the upper plate and the middle plate with the upper electrode facing the lower electrodes using Kapton tape (Fig. S1). Before bonding, the upper plate and middle plate are diced into 10 mm × 10 mm square shape with dicing saw. To assemble the ASIC with the system, wafer stacks with the upper plate and the middle plate are first flipped with the bottom of the middle plate facing up. The chip is then attached to the middle of the two pads on the middle plate with 5-minute epoxy. Several capacitors to work with the chip are also attached onto the region inside the coil using the same epoxy. Ultimately, wire bonding of gold is implemented to connect the chip, capacitors, coil, and sensor.

To complete the multilayer structure, the bottom plate is attached and aligned with the wafer stack and electronics. To bond the upper, middle, and lower plates together, a CO_2_ laser is utilized to dice the wafer stack into a circular chip using 100% power and 4% speed 6 times, simultaneously achieving localized layer-to-layer fusion bonding.

### Mechanical-electrostatic modeling

Finite element analysis (FEA) using commercial software (COMSOL) guided the optimization of the dimensions and sensor response of the system (Fig. S2). In the sensor simulation model, the upper plate consisted of a 200 μm thick, 5 mm diameter silica plate with a cavity of 4 mm in diameter and 3.6 μm in depth. The lower silica plate was 500 μm thick and 5 mm in diameter. The upper Ti electrode was 3.6 mm in diameter and 100 nm thick, with 100 nm thick oxide layer. The lower Ti electrode was 3.6 mm in diameter and 200 nm thick, with 30 nm thick oxide layer. The bottom surface of the sensor was fixed, and the upper and lower oxide layers were specified as contact surfaces. Young’s modulus and Poisson’s ratio of silica were 73.1 GPa and 0.17. Young’s modulus and Poisson’s ratio of Ti were 115.7 GPa and 0.321. The Young’s modulus and Poisson’s ratio of the oxide layers were 293.0 GPa and 0.27.

For the simulation, the sensor implanted under the skin, a 1-mm thick dermis layer with Young’s modulus of 1000 kPa and Poisson’s ratio of 0.48 and a 3-mm hypodermis layer with Young’s modulus of 50 kPa and Poisson’s ratio of 0.48 were built on top of the sensor (41) (Fig. S3). FEA using COMSOL also determined the sensor capacitance under certain environmental conditions, e.g., in liquid, which provided design parameters for the wireless transmission electronics.

### Direct force experiments

The force-capacitance relationship of the sensing system was assessed using a Bose Electroforce Dynamic Mechanical Analysis 3200. The instrument was fitted with an upper polyoxymethylene loading component 1.5 mm in diameter to apply normal force to the center of the sensor. Sensor powering and data readout for all tests was performed wirelessly via the base unit. The sensor was placed on top of the coil on the base unit. One end of the base unit was connected to a function generator and the other end was connected to an oscilloscope (Fig. S4). To approximate implanted conditions, the Bose test instrument was fitted with a flat surface acrylic loading component (10 mm × 10 mm square shape) that applied forces to a silicone layer (Ecoflex0030, 5 mm in thickness and 5 mm in diameter) overlying the sensor upper plate (Fig. 3A). Wireless sensor operation was the same as described above. In the presence of normal forces, both the silicone and sensor upper plate deformed.

### Hydrostatic pressure experiments

The sensing system was placed inside a 5 ml syringe which was filled with water (Fig. 3E). The base unit was set outside the syringe for wirelessly powering the sensing system and collecting data from the system. A digital pressure gage (MG1, SSI Technologies) was connected to the syringe to measure pressure as the syringe plunger was manually depressed to achieve target static pressures.

### Ex vivo experiments

The right hand of a rhesus macaque *(Macaca mulatta)* that had recently been euthanized for clinical reasons unrelated to this project was obtained. The experiment was performed within 18 h of death on the unfixed tissue. The hand was missing the thumb and index fingers. The sensor was implanted into the fingertip of the middle finger (Fig. 4A). The hand was then secured on top of a platform with a hole through which the middle fingertip was placed. A custom force-controlled device (H2W Technologies) with 6-axis force/torque sensor (Nano17, ATI Industrial Automation) was placed under the platform. It applied forces to the implanted fingertip via a 5-mm diameter plastic probe. The base unit was placed on the back of the hand centered on the middle fingernail for wirelessly powering and reading the sensor.

## Figures and Tables

**Table 1.** System specifications.

*Implantable sensor*	
Package diameter	6 mm
Package thickness	1.7 mm
Antenna diameter	4.8 mm
Antenna inductance	196 nH
Transmitter carrier frequency	35 MHz
Transmitter modulation format	On-off keying
Sensing resolution	3.4 fF @18pF
ASIC area	1.62 mm^2^
Power consumption	110.3 μW
*Wearable base unit*	
Antenna diameter	7.1 mm
Antenna inductance	206 nH
PCB area	43 mm × 37 mm
Sensor sampling rate	6.5 kS/s @18pF

## Data Availability

All data needed to evaluate the conclusions in the paper are available in the main text or the Supplementary Materials.
